# Modified ex utero intrapartum treatment procedure in a bicornuate uterus breech presentation Pierre Robin fetus with severe micrognathia and cleft palate

**DOI:** 10.1002/ccr3.1799

**Published:** 2018-09-12

**Authors:** Irit Duek, Ziv Gil, Ido Solt

**Affiliations:** ^1^ The Head and Neck Center Department of Otolaryngology Head and Neck Surgery Rambam Health Care Campus Haifa Israel; ^2^ Department of Gynecology and Obstetrics Rambam Health Care Campus Haifa Israel

**Keywords:** congenital airway obstruction, ex utero intrapartum treatment

## Abstract

Thorough prenatal evaluation allows for identification of fetuses with compromised airway. The ex utero intrapartum treatment procedure enables maintaining uteroplacental circulation during cesarean section while securing a potentially obstructed fetal airway, converting a potentially catastrophic situation into a controlled one. An expert multidisciplinary team is the key to success.

## Graphical abstract

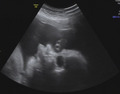

## 1 INTRODUCTION

Advances in the prenatal imaging and diagnosis of congenital head, neck, and thorax pathologies and malformations have allowed for great improvements in the outcome of fetuses at risk of hypoxic brain injury and mortality during delivery due to potential airway obstruction.^1^ The ex utero intrapartum treatment (EXIT) procedure is currently considered the gold standard for delivering fetuses with congenital airway obstruction and cardiorespiratory compromise. It permits controlled securing of the fetal airway while maintaining placental circulation and oxygen support, converting an emergent life‐threatening, potentially catastrophic situation into an elective and controlled one. The EXIT procedure is defined as a procedure performed after partial externalization of the fetus from the uterine cavity, while maintaining uterine volume and fetoplacental circulation, ensuring the fetal oxygenation during airway access. Once adequate airway and ventilation are secured, the fetus can be safely taken off placental support.^2^ The indications for EXIT procedure include various conditions associated with possible fetal airway obstruction (Table 1, 2, 3, 4, 5, 6). The successful outcome of an EXIT procedure rests upon thorough and accurate prenatal investigation, appropriate presurgical planning, and the availability of a highly skilled multidisciplinary team and facilities. In this paper, we present a case of a fetus with severe micrognathia, cleft palate, and Pierre Robin sequence (PRS), which was diagnosed prenatally in a bicornuate uterus and breech presentation. Antenatal multidisciplinary team planning and treatment were instituted to ensure a secure airway at delivery, utilizing a modified EXIT procedure, avoiding a potentially catastrophic outcome.

**Table 1:** Indications for EXIT procedure

*Fetal cervical masses*—cervical teratoma, hemangioma, cervical lymphangioma (cystic hygroma), hamartoma, neuroblastoma, congenital goiter, parotid masses, branchial cleft cyst, thyroglossal duct cyst, congenital thyroid tumor, laryngocele, nuchal edema, choristoma, neural tube defects (eg, cervical myelomeningocele), lipoma
*Lung anomalies*—congenital cystic adenomatoid malformation (CCAM), congenital pulmonary airway malformation, bronchopulmonary sequestration
*Mediastinal masses*—teratoma, lymphangioma
*Micrognathia*
*Congenital high airway obstruction syndrome (CHAOS)*—tracheal or laryngeal atresia
*Reversal of tracheal clipping*—congenital diaphragmatic hernias (CDH)
*Exit‐to‐ECMO* (extracorporeal membrane oxygenation)—congenital heart anomalies
*Exit‐to‐separation for conjoined twins*

CCAM, congenital cystic adenomatoid malformation; CDH, congenital diaphragmatic hernias; CHAOS, congenital high airway obstruction syndrome; ECMO, extracorporeal mechanical oxygenation; EXIT, ex utero intrapartum treatment.

## 2 CASE PRESENTATION

**Figure 1:**
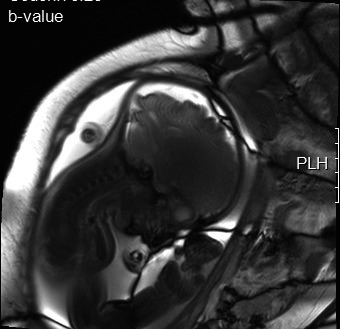
Prenatal fetal MRI at 32 + 2 wk of gestation

**Figure 2:**
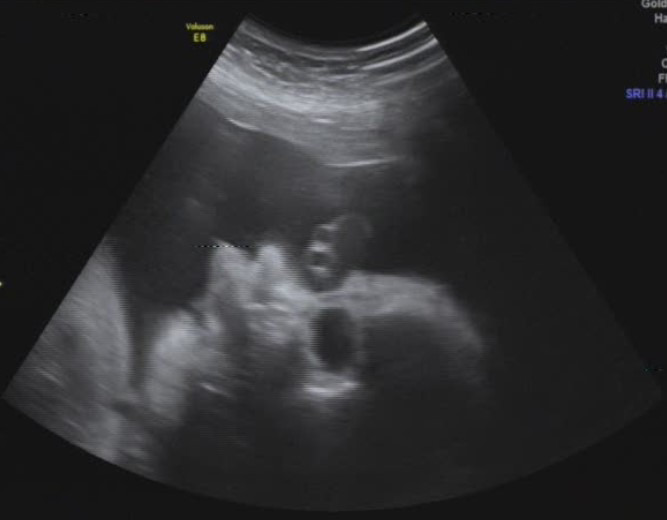
Prenatal fetal US at 35 + 2 wk—Appropriate for gestational age fetus. Polyhydramnios, retrognathia‐micrognathia, dropped tongue, and posterior pharynx were demonstrated. A palate was not demonstrated. Findings may be compatible with Pierre Robin sequence

**Figure 3:**
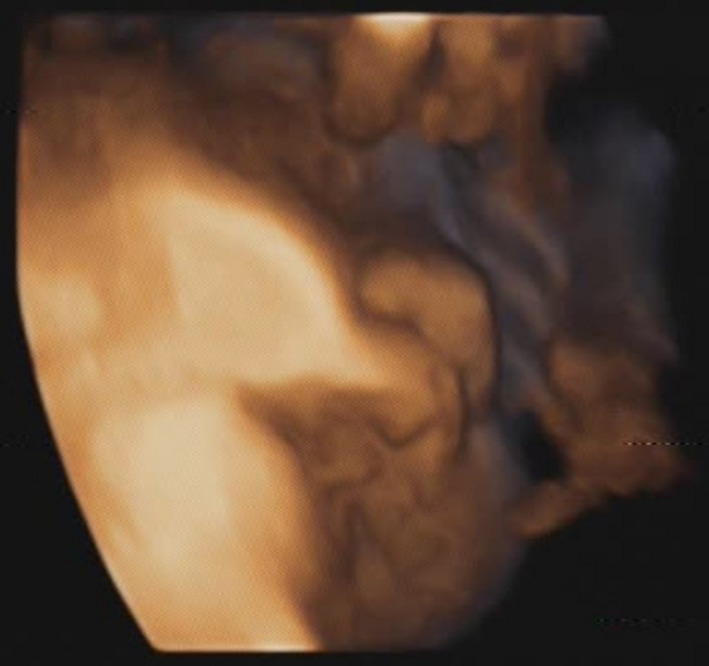
Prenatal fetal three‐dimensional ultrasound imaging at 35 + 2 wk of gestation further demonstrating the degree of micrognathia and retrognathia present

**Figure 4:**
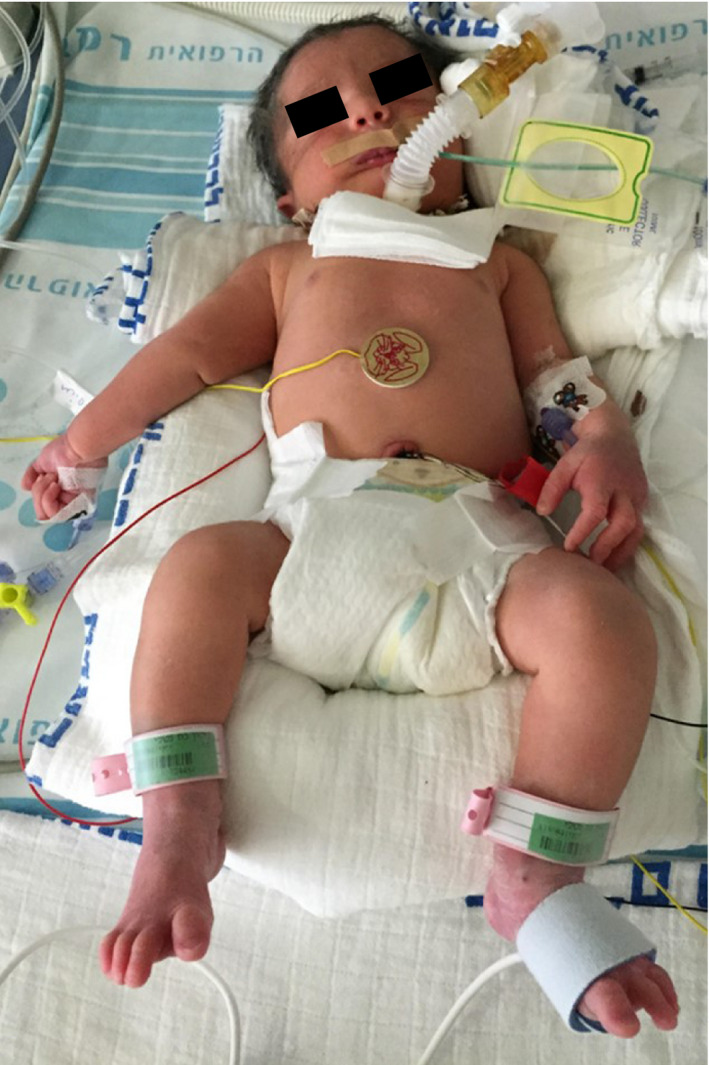
Tracheostomy‐dependent neonate with severe micrognathia and associated Pierre Robin sequence, at day 1 after delivery

**Figure 5:**
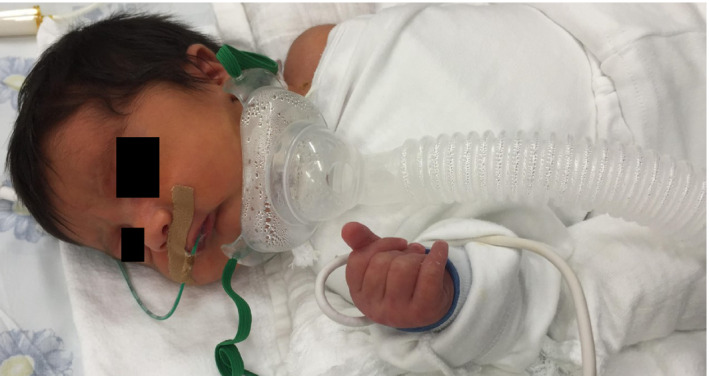
The neonate at day 6 after delivery

A 31‐year‐old, gravida 4, para 1, woman was referred to our institute due to suspected fetal Pierre Robin sequence, at 34 + 6 weeks of gestation. A difficult intubation was anticipated, and the possibility of achieving surgical airway in an EXIT procedure was planned. The woman, which is known to have a bicornuate uterus, had previously undergone a cesarean section during her first labor due to breech presentation at 39 weeks of gestation. The child was born weighing 2710 g with micrognathia and cleft palate, not requiring an EXIT procedure. She also underwent two spontaneous abortions at 8 weeks of gestation. A prenatal magnetic resonance imaging (MRI) at 32 + 2 weeks of gestation (Figure 1) and an ultrasound (US) demonstrated severe micrognathia. Fetal echocardiography and genetic consultation were normal. Amniocentesis was not performed due to maternal refusal. At 35 + 2 weeks of gestation, a two‐dimensional US (Figure 2) was repeated and a three‐dimensional US was performed (Figure 3) to evaluate in more detail the fetal anatomy and growth. US showed polyhydramnios, dropped tongue, posterior pharynx, and retrognathia‐micrognathia. No palate was observed. While the delivery was planned to 37 weeks of gestation (for fetal lung maturity),^7^ at 35 + 3 weeks of gestation, the patient started feeling regular uterine contractions, and it seemed as she was going into spontaneous labor. Contraction stress test was negative, US showed breech presentation, and the blood pressure and pulse were within normal ranges. After a multidisciplinary discussion, the decision was made to deliver through a cesarean section with preparation for a possible EXIT procedure. Members of the team (including fetomaternal medicine/obstetricians, otolaryngologist, neonatologists, anesthesiologist, pediatric anesthesiologist, pediatric pulmonologist, midwifery, and neonatal intensive care unit nursing) were gathered to prepare for the delivery and perform an EXIT procedure to secure an airway if necessary. Due to breech presentation, bicornuate uterus (pregnancy in the left uterus), placental location (fundal posterior), low uterine segment dehiscence, and umbilical cord entanglement around the fetus neck (two complete loops), a classic EXIT procedure could not be performed. The plan was that after the delivery of the breech presentation newborn, the placenta would be left attached supplying oxygen to the newborn. Uterine muscle relaxants will be given to provide maximum time of placental oxygenation to the newborn to allow the otolaryngologist to secure temporary newborn airway. After pulsation will not be felt in the umbilical cord, it would be transected, and later, definitive airway will be established. Through a cesarean section, the baby which was not crying nor breathing spontaneously was delivered and put at a sterile table at the mother's right side with the umbilical cord intact utilizing a modified EXIT procedure. The total maternal blood loss did not exceed 1000 mL, and there were no signs of maternal hemodynamic compromise during the procedure or maternal postoperative decrease in hemoglobin values. A live 2975 g female infant with Apgar scores of 6 and 6 at 1 and 5 minutes, respectively, was born (appearance—2, pulse—2, grimace—1, activity—1, respiration—0). Not being able to breathe spontaneously, the baby demonstrated signs of upper airway obstruction. Initially, the team waited for signs of spontaneous breathing before any intervention; however, the neonate did not seem to be able to breathe on her own. Only then, when it was clear that airway support is essential for saving the baby's life, attempts for direct laryngoscopy were undergone. After two failed direct laryngoscopy attempts by the pediatric anesthesiologist, another attempt was made by the otolaryngologist, and since a direct clear vision of the larynx could not be achieved due to the severe micrognathia, a decision to perform a tracheotomy to ventilate and secure the neonate airway was made. A laryngeal mask was inserted, and then, a tracheotomy was performed. Blood gases from the umbilical cord showed pH level of 7.3. Birthweight was 2975 g. The neonate was transferred to the neonatal intensive care unit, was ventilated through a 3.0 neonatal Shiley tracheostomy tube, and was stable (Figure 4). Cardiac echocardiography, abdominal US, and brain US were normal. The neonate was successfully disconnected from the ventilator 4 days after delivery and started breathing spontaneously (Figure 5). Long‐term airway management was planned, including distraction osteogenesis to attempt an improvement of airway dynamics, as well as cleft palate repair.^7^ At the age of 2 years, the toddler is healthy and achieved all the developmental milestones on time.

## 3 DISCUSSION

Pierre Robin sequence is relatively common, occurring in approximately 1 in 8500 births, and it is heterogeneous in etiology. Infants with Pierre Robin sequence usually present with airway obstruction. Anatomically, the larynx lies under the base of the tongue in patients with micrognathia and glossoptosis making direct laryngoscopy or intubation difficult and occasionally impossible.^8^, ^9^ When a fetus is diagnosed antepartum with anatomic congenital anomalies which may compromise the airway during delivery, utilizing EXIT procedure with coordinated team management during delivery should be considered. For the appropriate indication, when airway obstruction is anticipated during delivery, the EXIT procedure is considered the standard of care. In a recently published paper, Kornacki et al^10^ reported their experience with three successful EXIT procedures performed in fetuses with neck tumors (thyroid goiter, neck lymphangioma, and giant neck teratoma). Early evaluation and planning by a multidisciplinary team, based upon thorough assessment of the fetal anatomy with fetal two‐ and three‐dimensional US, echocardiography, and MRI, are essential to correctly qualify the fetus for the EXIT procedure.^2^, ^3^, ^5^, ^7^, ^8^, ^9^, ^10^ As opposed to the cesarean section deliveries where uterine contraction is desired to reduce maternal blood loss, during the EXIT procedure the achievement of tocolysis and uterine hypotonia are essential to preserve uteroplacental circulation and prevent placental separation,^2^ increasing the risk of massive maternal blood loss. Placental location should also be considered since an anterior low placenta may limit the incision (and thus the position for the delivery of the fetal head). In a traditional EXIT procedure, after hysterotomy, the fetal head is rotated in an occipitoposterior position on delivery. The fetal head and neck and one arm are delivered, and the rest of the fetal body and umbilical cord remain in the uterus to preserve heat and as much amnion as possible to limit amniotic fluid loss. The case presented herein is unique and especially challenging due to various obstetrical factors, including breech presentation, bicornuate uterus (pregnancy in the left uterus), placental location (fundal posterior), low uterine segment dehiscence, and umbilical cord entanglement around the fetus neck (two complete loops), making it impossible for a classic EXIT procedure to be performed, necessitating a creative modified solution.

## 4 CONCLUSION

Prenatal diagnosis of fetal craniomaxillofacial or cervical malformation obstructing the upper airway is essential to reduce perinatal morbidity and mortality, allowing for maternal‐fetal monitoring during pregnancy and defining the best approach for delivery. EXIT procedure is indicated almost in any situation in which the fetal airway might be compromised during delivery. With the use of the EXIT procedure, congenital malformations obstructing the airway that were previously fatal during delivery can be managed with a semielective procedure that allows time for securing the airway by direct laryngoscopy or fiberoptic and orotracheal intubation or tracheostomy before fetoplacental separation. In the presented case, the intervention success was not only due to the early diagnosis of malformation responsible for the fetal airway obstruction, but also fundamentally to the planning of the procedure by a multidisciplinary team of neonatologists, pediatric pulmonologists, obstetricians, anesthesiologists, and otolaryngology‐head and neck surgeons.

## CONFLICT OF INTEREST

None for either author.

## AUTHOR CONTRIBUTIONS

ID: wrote the manuscript, conceived and designed the study, and participated in patient's management and follow‐up. ZG: managed the case from the otolaryngology point of view, securing the neonate airway, planned and performed the operation on the baby. IS: managed the case, from first intrauterine diagnosis and decision making, presented the case to the multidisciplinary team, planned and performed the operation on the mother, delivered the child.
